# Implementing polymeric pseudorotaxanes for boosting corneal permeability and antiaspergillus activity of tolnaftate: formulation development, statistical optimization, ex vivo permeation and in vivo assessment

**DOI:** 10.1080/10717544.2022.2094499

**Published:** 2022-07-10

**Authors:** Diana Aziz, Sally Mohamed, Saadia Tayel, Amal Makhlouf

**Affiliations:** a Faculty of Pharmacy, Department of Pharmaceutics and Industrial Pharmacy, Cairo University, Cairo, Egypt; b Faculty of Pharmacy, Department of Microbiology and Immunology, Cairo University, Cairo, Egypt

**Keywords:** Keratitis, Tolnaftate, ocular permeability, susceptibility, Aspergillus niger

## Abstract

Fungal keratitis (FK) is a devastating ocular disease that can cause corneal opacity and blindness if not treated effectively. Tolnaftate (TOL) is a selective fungicidal drug against Aspergillus spp. which are among the most common causes of mycotic keratitis. TOL is lipophilic drug with low water solubility and permeation which act as obstacles for its clinical ocular efficacy. Hence, this study aimed to statistically optimize a novel polymeric pseudorotaxanes (PSRs) containing TOL for enhancing its ocular permeability and antifungal effect. For achieving this goal, a full 3^1^.2^2^ factorial design was fashioned for preparing and optimizing TOL-PSRs using film hydration technique. Three formulation variables were studied: drug amount (X_1_), weight ratio of Pluronics to HPβCD (X_2_) and Pluronic system (X_3_). Entrapment efficiency percent (EE%) (Y_1_), particle size (PS) (Y_2_) and zeta potential (ZP) (Y_3_) were set as dependent variables. The selected optimal TOL-PSRs (PSR1) showed EE% of 71.55 ± 2.90%, PS of 237.05 ± 12.80 nm and ZP of −32.65 ± 0.92 mV. In addition, PSR1 was compared to conventional polymeric mixed micelles (PMMs) and both carriers significantly increased the drug flux and resulted in higher amount permeated per unit area in 8 h compared to drug suspension. The histopathological studies assured the safety of PSR1 for ocular use. The in vivo susceptibility testing using Aspergillus niger confirmed that PSR1 displayed sustained antifungal activity up to 24 h. The obtained results revealed the admirable potential of PSR1 to be used as novel nanocarriers for promoting TOL ocular delivery.

## Introduction

Fungal keratitis (FK) is one of the devastating ocular diseases that can cause corneal opacity and blindness if not diagnosed and treated effectively (Li et al., 2018). Trauma, immunosuppressive diseases, topical steroid use, chronic keratitis, corneal surgery and contact lenses extended wear are the leading pathogenic factors of FK (Abdelbary et al., 2016). It is evident that Aspergillus and Fusarium species are the most common causative organisms of FK (Acharya et al., 2017). Furthermore, Candida has been considered the major frequent cause of FK in template climates and resulted in the worst clinical outcome (Nielsen et al., 2015). Clinically, FK presents with ocular pain, blurred vision and foreign body sensation.

The ultimate goal in FK treatment is to retard infection diffusion, minimize complication, prevent future recurrence and consequently conserve vision. This could be achieved by distinctive diagnostic procedures that can correctly identify disease process concurrently with administration of the effective antifungal therapy immediately upon diagnosis (ElMeshad & Mohsen, 2016; Li et al., 2018). Systemic antifungals are not the first line treatment for FK as it requires high doses to achieve the therapeutic concentrations at the target ocular tissue which may result in unwanted side effects (Fetih, 2016). Hence, topical antifungals which formulated as conventional eye drops are considered as the gold standard treatment for FK due to their rapid onset of action, simplicity of instillation, low cost, noninvasiveness and better patient compliance (Younes et al., 2018). However, the efficacy of the current antifungal therapies is limited due to drug insensitivity, poor drug availability, emergence of resistant strains and lack of effective ocular drug delivery system (Liu et al., 2013; Li et al., 2018). There are three main groups of antifungal drugs that are currently used for treatment of FK, namely, polyenes (amphotericin B, nystatin and nantamycin), azoles (imidazoles and triazoles) and flucytosine (5-flurocytosine) (Kaur et al., 2008). However, the poor penetration, stability and solubility of these drugs have limited their use in FK treatment (Li et al., 2018).

Furthermore, the efficacy of antifungal therapies through this route is limited by several physiological and anatomical barriers of human eyes; including blinking reflex which is responsible for removal of about 95% of the applied dose, the tear film barrier, ocular surface small capacity, induced lacrimation, low corneal permeability and enzymatic degradation of the drugs by the anterior segment enzymes (Kakkar & Kaur, 2011; Emad Eldeeb et al., 2019). Hence, good corneal penetration and prolonged residence of the drug delivery system in the cornea is the main prerequisite to overcome these barriers and enhance ocular drug delivery (Li et al., 2018). The development of nanoformulations provides a new tool to overcome ocular barriers, improve drug retention time on the corneal surface and enhance drug permeability and bioavailability compared to conventional eye products (Khiev et al., 2021). Therefore, the design of novel nanosystems, including elastic vesicles (spanlastics, transferosomes), solid lipid nanoparticles, microemulsions and mixed micelles has become inescapable for the continuous development in the ocular pharmaceutical industry.

Tolnaftate (TOL) is a synthetic thiocarbamate antifungal agent acts selectively by inhibiting squalene epoxidase, an important microsomal enzyme in biosynthetic pathway of ergosterol (an essential component of the fungal membrane). Thus, squalene is accumulated in the cell wall of the fungi and ergosterol is depleted which affects membrane permeability, membrane-bound enzymes and growth causing cell death (Abdelbary et al., 2016; Abousamra & Mohsen, 2016). Furthermore, it is active against filamentous fungi e.g., Aspergillus spp. which are among the most common causes of mycotic keratitis. Aspergillus, if not early diagnosed, can also cause reduction in visual capacity due to its early macular involvement, choroidal damage and retinal necrosis (Spadea & Giannico, 2019). Hence, TOL is expected to be a promising candidate for the treatment FK due to its selective fungicidal properties, intermediate molecular mass (307.4) and lipophilicity (log P 5.5) which facilitate its penetration across the corneal lipid rich epithelial and endothelial cell membrane (Kaur et al., 2008). However, being a biopharmaceutical classification system (BCS) IV drug, TOL possesses low aqueous solubility (0.00054 mg/mL) and penetration which act as obstacles that limit its clinical ocular efficacy (Akhtar et al., 2016). Hence, the challenge is to formulate TOL in an appropriate delivery system to enhance its aqueous solubility and consequently promoting its ocular permeation and retention.

Recently, polymeric mixed micelles (PMMs) have gained increased attention as a talented nanotechnology- based ocular delivery system. PMMs are nanoscopic structure designed by self-assembly of two or more amphiphilic polymers such as Pluronics. They consist of an inner hydrophobic core capable of harboring lipophilic drugs with poor aqueous solubility through hydrophobic interaction and an outer hydrophilic shell serving as a stabilizing interface that insolates the encapsulated substances from the external aqueous media (Nour et al., 2016; Younes et al., 2018). PMMs have the advantages of non-irritancy, biodegradability, thermodynamic stability and ability of controlling drug release (Yokoyama, 2014). Furthermore, PMMs are characterized by their superior drug loading and solubilizing capacity compared to regular single micelles, owing to their relatively larger core (Abd-Elsalam et al., 2018). It has been also demonstrated that PMMs could enhance the ocular delivery of poorly water soluble drugs e.g., cyclosporine (Grimaudo et al., 2018), myricetin (Hou et al., 2019) and curcumin (Duan et al., 2015) via enhancing their corneal permeability and retention.

Cyclodextrins (CDs) complexation with poorly soluble drugs is also one of the most common approaches that could enhance drug stability and solubility in aqueous media (Nogueiras-Nieto et al., 2009). Natural CDs include three well known cyclic oligosaccharide, namely, α, β and γ. Among these, β-CD is the ideal one for complexation due to its perfect cavity size, efficient drug loading and relatively low cost (Gidwani & Vyas, 2015). Furthermore, β-CD can also improve the drug ocular bioavailability by enhancing its permeation through the ocular aqueous tear film and bio-membrane (Jansook et al., 2016). Recently, various novel molecular structures have been obtained via inclusion of polymer chains into CDs cavities (Folch-Cano et al., 2014). The use of CDs was found to enhance the properties of the used polymer, and on the other hand the use of polymers also enhances the solubilizing action of CDs in a given dosage form due to the formation of polymer/CD inclusion complex in which both polymers and CD contribute synergistically (Loftsson & Brewster, 1996). Polymer/CD inclusion complex are called polymeric pseudorotaxanes (PSRs) in which many CD molecules are threaded into a single polymer chain (Folch-Cano et al., 2014). PSRs are novel nanosystems designed for safe and efficient delivery of a wide range of practically insoluble hydrophobic drugs (Sayed et al., 2018). Nogueiras-nieto et al. described PSRs as self-assembled structure formed between Pluronics and CDs where the initially formed pseudorotaxanes are associated together forming soluble complexes (Nogueiras-Nieto et al., 2009). In another words, polymer chains of Pluronics fit into the hollow cavity of CDs truncated cone forming soluble PSRs complexes (Nogueiras-Nieto et al., 2012). It is generally accepted that PSR complex formation between host CDs and guest polymers proceeds mainly in three steps, 1: the polymer fits into CD cavity to generate hydrophilic complexes, 2: the threaded CD molecules are stacked partially along polymer axis and 3: precipitation of PSRs by the intermolecular hydrophobic interactions between CD molecules in the complexes (Nogueiras-Nieto et al., 2009). In PSRs, the formed inclusion complex can be dissociated to its forming molecules in a dynamic equilibrium (Nogueiras-Nieto et al., 2009). Hence, the simultaneous coexistence of PSR complex and its forming molecules lead to enhanced drug solubilization and permeability using reduced proportion of each solubilizing agent due to synergistic effect (Nogueiras-Nieto et al., 2009; Sayed et al., 2018). However, the extent of the synergism depends strongly on the nature and the concentration of both CD and the used surfactant. Hence, stringent effort should be carried out for investigating and optimizing the characteristics of these nanocarriers to act as an ocular delivery platform.

Thus, our work aims firstly to devise a novel TOL-PSRs for enhancing the aqueous solubility and subsequently corneal permeation of TOL according to 3^1^.2^2^ full factorial design in order to study the effect of different variables on the PSRs characteristics and to elucidate the optimal formulation. Secondly, to confirm the hypothesized enhanced ocular permeation potential of PSRs, ex vivo permeation studies of the optimal PSRs was conducted in comparison to drug suspension. Furthermore, the in vivo anti fungal efficacy of the optimal PSR was studied and compared to drug suspension. Finally, in vivo histopathological studies were done to confirm the ocular safety and tolerability of the prepared optimal PSR.

## Materials and methods

### Materials

Tolnaftate (TOL) was granted by Hikma pharmaceuticals (Cairo, Egypt). Pluronic F 127 (F127), Pluronic L 121 (L121), Pluronic P 123 (P 123), hydroxyl propyl β-Cyclodextrin (HPβCD) and acetonitrile (HPLC grade) were purchased from Sigma Chemical Co. (St. Louis, MO, USA). Methanol was purchased from El-Nasr Pharmaceutical Chemicals Co. (Abu-Zaabal, Cairo, Egypt).

### Preparation of TOL polymeric pseudorotaxanes (TOL-PSRs)

TOL-PSRs were primed using film hydration technique (Younes et al., 2018) by varying TOL dose (10 or 20 mg), Pluronic system (200 mg in ratio 1:1 of F127/P123 or F127/L121 or P123/L121) and using different Pluronics to HPβCD weight ratio (1:1 and 1:0.5). Briefly, specified amounts of TOL and Pluronic system were weighed and dissolved in 10 mL acetonitrile in a round-bottom flask (250 mL). The solvent was then slowly evaporated under reduced pressure at 60 °C using rotary evaporator (Rotavapor, Heidolph VV 2000, Burladingen, Germany) for 30 min at 90 rpm to obtain thin dry film of components on the inner wall of the flask. The formed dried film was then hydrated with 10 mL of ultra-pure distilled water containing different amount of HPβCD (100 or 200 mg) by rotating the flask in a water bath maintained at 30 °C for 1 h at 210 rpm using the same apparatus under normal pressure to form pure colloidal dispersion of TOL-PSRs. Finally, the resultant fine-tuned dispersions were left to equilibrate overnight at 4 °C for further characterization.

### Experimental design

To investigate the influence of formulation variables on PSRs characteristics, 3^1^.2^2^ factorial design was fashioned for preparing TOL-PSRs. The statistical procedure was performed using Design-Expert® software (Version 7, Stat-Ease, Inc., Minneapolis, MN, USA) for analyzing the experimental trials and estimating the optimal formulation based on desirability function. According to the employed factorial design, 12 formulae were prepared. The independent variables were: drug amount (X_1_), weight ratio of Pluronics to HPβCD (X_2_) and Pluronic system (X_3_). Entrapment efficiency percent (EE%) (Y_1_), particle size (PS) (Y_2_) and zeta potential (ZP) (Y_3_) were set as dependent variables. Analysis of variance (ANOVA) was performed for estimating the significance of the model and terms. Probability p-values (*p* < 0.05) denoted significance. For the type of Pluronic system (X_3_), post-hoc analysis was performed using Tukey’s honest significant difference (HSD) test using SPSS software 17.0 (SPSS Inc., Chicago, IL).The factors studied and their respective levels are shown in Table 1. The design matrix including the composition of TOL-PSRs along with their characterization is shown in Table 2.

**Table 1.** Full factorial design (3^1^.2^2^) used for optimization of TOL-PSRs.

Factors (independent variables)		Levels	
X_1_: Drug amount (mg)	10		20
X_2_: Weight ratio of Pluronics to HPβCD	1:1		1:0.5
X_3_: Pluronic system	F127/P123	F127/L121	P123/L121
Responses (dependent variables)		Desirability Constraints	
Y_1_: EE%		Maximize	
Y_2_: PS (nm)		Minimize	
Y_3_: ZP (mV)		Maximize (as absolute value)	

Abbreviations: EE%, entrapment efficiency percent; PS, particle size; ZP, zeta potential; TOL-PSRs, tolnaftate polymeric pseudorotaxans.

**Table 2.** Experimental runs, independent variables, and measured responses of the 3^1^.2^2^ full factorial experimental design of TOL-PSRs compared to conventional PMMs.

Formula code	X_1_Drug amount (mg)	X_2_Weight ratio of Pluronics to HPβCD	X_3_Pluronic system	EE% ^a^	PS (nm)^a^	PDI ^a^	ZP (mV)^a^
PSR1	10	1:1	F127/L121	71.55 + 2.90	237.05 ± 12.80	0.45 ± 0.05	−32.65 ± 0.92
PSR2	10	1:1	F127/P123	85.68 ± 3.54	387 ± 13.44	0.56 ± 0.11	−18.80 ± 5.37
PSR3	10	1:1	P123/L121	94.05 ± 1.91	560.65 ± 1.34	0.61 ± 0.08	−29.25 ± 0.78
PSR4	10	1:0.5	F127/L121	79.45 ± 3.04	247.45 ± 10.96	0.65 ± 0.10	−29.80 ± 0.28
PSR5	10	1:0.5	F127/P123	81.88 ± 2.43	302.75 ± 3.89	0.32 ± 0.02	−19.20 ± 0.85
PSR6	10	1:0.5	P123/L121	91.20 ± 0.71	773.65 ± 36.27	0.52 ± 0.22	−26.85 ± 1.91
PSR7	20	1:1	F127/L121	20.50 ± 0.71	273.55 ± 3.18	0.53 ± 0.01	−33.30 ± 4.53
PSR8	20	1:1	F127/P123	12.35 ± 4.45	601.50 ± 2.12	0.57 ± 0.13	−22.00 ± 2.68
PSR9	20	1:1	P123/L121	23.50 ± 4.95	395.70 ± 23.33	0.45 ± 0.16	−23.15 ± 1.06
PSR10	20	1:0.5	F127/L121	15.10 ± 2.55	264.55 ± 31.75	0.55 ± 0.16	−24.80 ± 0.71
PSR11	20	1:0.5	F127/P123	10.61 ± 0.86	428 ± 138.73	0.63 ± 0.08	−24.60 ± 1.13
PSR12	20	1:0.5	P123/L121	16.95 ± 0.35	463.35 ± 26.38	0.63 ± 0.19	−25.95 ± 0.64
Conventional PMMs	10	1:0	F127/l121	50.24 ± 1.89	254.35 ± 66.11	0.29 ± 0.04	− 30.60 ± 1.13

Abbreviations: EE%, entrapment efficiency percent; PS, particle size; PDI, polydispersity index; ZP, zeta potential; TOL-PSRs, tolnaftate polymeric pseudorotaxans; PMMs, polymeric mixed micelles.

^a^ Data represented as mean ± SD (*n* = 3).

### In vitro characterization of TOL-PSRs

#### Determination of TOL entrapment efficiency percent (EE%)

EE% of TOL in PSRs was determined from the dispersion obtained after separation of the unentrapped drug by filtration through Whatman filter paper (grade No. 1, 11 μm) (Fahmy et al., 2018). One mL of the separated PSRs were disrupted by sonication with methanol and the concentration of the entrapped TOL was measured spectrophotometrically (Shimadzu, model UV-1601PC, Kyoto, Japan) by measuring the ultraviolet (UV) absorbance at the predetermined λ_max_ of TOL in methanol (257 nm) (Abousamra & Mohsen, 2016). Each result was the mean of three determinations ± standard deviation (SD). Drug EE% was determined using the following equation:

$$EE\%=\frac{Incorporated\;amount\;of\;TOL}{Total\;amount\;of\;TOL}\times 100$$

#### Determination of particle size (PS), polydispersity index (PDI), and zeta potential (ZP)

The mean PS, PDI, and ZP of the prepared TOL PSRs were analyzed by Zetasizer Nano ZS (Malvern Instrument Ltd., Worcestershire, UK) based on dynamic light scattering technique. Before each measurement, the prepared dispersions were properly diluted with de-ionized water in order to obtain the suitable scattering intensity. ZP measurement was performed using the same instrument for observing the electrophoretic mobility of the particles in the electric field. Three replicates were taken for each sample.

### Optimization of TOL-PSRs

Design-Expert^®^ software was employed to obtain the optimal TOL-PSR formula by implementing restrains on EE%, PS, and ZP as shown in Table 1. The formula of the highest desirability value (nearest to 1) was selected for further investigation. For checking the validity of the predicted responses given by the software, the selected PSR formula was prepared and compared with the predicted responses (Fares et al., 2018). The percentage error for each dependent variable (EE%, PS and ZP) was also calculated from the following equation:

$$\mathrm{Percentage}\;\mathrm{error}=\frac{\mathrm{Observed}\;\mathrm{values}-\mathrm{Predic}ted\;\mathrm{values}}{\mathrm{Observed}\;\mathrm{values}}\times 100$$

### Transmission electron microscopy (TEM)

The morphological aspects of the optimal formulation were assessed using TEM. One drop of the undiluted dispersion was placed on a carbon coated copper grid and allowed to adhere for about 1 min. The dispersion in excess was then removed using a tip of filter paper. Samples were left to dry for 10 min at room temperature for investigation (Khalil et al., 2017). Finally, the air dried sample was examined under Joel TEM (Joel JEM 1230, Tokyo, Japan) and photographed (Fares et al., 2018).

### Differential scanning calorimetry (DSC)

Thermal analysis was performed for the pure drug, Pluronic system, HPβCD, freeze dried optimal formula and physical mixture of TOL with its components using differential scanning calorimeter (Shimadzu DSC-60, Shimadzu Corp., Kyoto, Japan) calibrated with purified indium (99.9%). Approximately 5 mg of each sample was placed in standard aluminum pans, crimped and heated in a temperature range of 10–300 °C at a scanning rate of 10 °C/min with continuous purging of nitrogen (25 mL/min).

### Preparation of conventional polymeric mixed micelles (PMMs)

For investigating the impact of incorporating CDs on the micellar physico-chemical properties, conventional PMMs was prepared by the previously mentioned manner utilizing the same components of the optimal PSR without incorporating HPβCD and then evaluated with respect to EE%, PS, and ZP. The obtained results were then statistically analyzed by Student’s t-test using SPSS software 17.0. Difference at *P* ≤ 0.05 was considered significant.

### In vitro release

The release of TOL from the optimal PSRs and the conventional PMMs was tested using membrane diffusion technique (Abdelbary & Tadros, 2013). Due to the low aqueous solubility of TOL, the experiment was performed in methanol/water mixture in the ratio of 3:2 as the release medium to maintain sink condition (Nour et al., 2016). Based on the predetermined EE%, accurate volume of the filtered PSR and PMMs (containing 300 µg TOL) was placed in a presoaked dialysis bag (Spectra/Por^®^ 12,000-14,000 molecular weight cutoff, Spectrum Laboratories Inc., USA). The bag was then immersed in amber colored bottle containing 100 mL of the release medium. The temperature was maintained at 37 °C with continuous stirring at 100 rpm using a magnetic stirrer (MSH-20D Hotplate Stirrer Unit). Aliquots of 3 mL were periodically withdrawn from the release medium at specified time intervals (0.25, 0.5, 1, 2, 4, 6, 8 h) and compensated by an equal volume of the release medium (Aziz et al., 2018). Samples were then analyzed spectrophotometrically at 257 nm. The experiment was done in triplicate and the mean values of percentage drug released (± SD) were plotted versus time. TOL aqueous suspension (1 mg/1 mL) was considered as a reference and its release was performed simultaneously in a similar way (Kakkar & Kaur, 2011). The obtained drug release profiles were adopted to zero order, first order and Higuchi diffusion model (Higuchi, 1963). The model of the highest coefficient of determination (R^2^) was considered the most fitting. T_50%_, time required for releasing 50% of the loaded TOL, was calculated based on the best fitting equation showing the highest R^2^ (Abdelrahman et al., 2017). Furthermore, to study the effect of formulation variables on the rate and extent of TOL release, 3 other PSR formulae (PSR2, PSR4 and PSR5) were selected and their release profiles were also investigated in a similar way and statistically analyzed with respect to T_50%_ and the amount released after 8 h (Q8h). These formulations were selected for different reasons. First, they have the same amount of TOL (10 mg) with different compositions (Pluronics to HPβCD weight ratio and Pluronic system). Second, they have close PS and EE% (Table 2). Hence, they represent a good model of the prepared PSRs that is needed to study the effect of formulation variables on TOL release.

### Ex vivo studies

#### Corneas preparation

The study protocol was revised and approved by the Research Ethics Committee, Faculty of Pharmacy, Cairo University, Egypt (PI 2982). Corneas used in this study were obtained from adult male New Zealand albino rabbits weighing 2.5–3.0 kg. The rabbits were anesthetized with an intramuscular injection of an anesthetic agent; ketamine 35 mg/kg, and a relaxing agent; xylazine 5 mg/kg (Eldeeb et al., 2019). The animals were then euthanized by decapitation. The eyes were immediately enucleated and the corneas were excised from the globes with a small scleral portion for easy mounting. The excised corneas were cautiously rinsed with saline and checked for being intact without wrinkles or pores before mounting. The transparent corneas were used in the permeation experiment within 30 min of animals’ scarification (Kakkar & Kaur, 2011).

#### Corneal permeation study

The excised corneas were sandwiched between the donor and receptor compartment of static vertical Franz diffusion cell (0.64 cm^2^). Optimal TOL-PSRs formula, conventional PMMs and TOL suspension (all equivalent to 300 µg TOL) was loaded into the donor compartment on the corneal surface under non-occlusive condition. The release medium (100 mL of methanol: water (3:2)) was kept in the receptor compartment under continuous stirring (100 rpm) at temperature of 37 °C (Sayed et al., 2018). At predetermined time intervals (0.5, 1, 2, 4, 6 and 8 h), samples (3 mL) were taken from the receptor compartment and immediately replaced with equal volume of fresh medium. The experiment was conducted in triplicate and the results were presented as mean ± SD. The amount of TOL in the withdrawn samples was then determined using a validated HPLC method. The cumulative amount of TOL permeated per unit area (µg/cm^2^) was plotted against time (h). The flux (J_max_) at 8 h and the enhancement ratio (ER) were calculated from the following equations (El Zaafarany et al., 2010).

$$J_{\max}=\frac{Amount\;of\;drug\;permeated}{Time\times \;Area\;of\;membrane}$$

$$ER=\frac{J_{\max\;}of\;the\;optimal\;nano\;formulation}{J_{\max\;}\;of\;the\;drug\;suspension}$$

The differences in flux values as well as total amount of TOL permeated were statistically analyzed by one-way ANOVA using SPSS software 17.0. Post-hoc analysis was performed using Tukey’s HSD test. Difference at *p* ≤ 0.05 was considered significant.

#### HPLC determination

A validated isocratic reported HPLC method was used for quantification of TOL with some modifications (Kezutyte et al., 2010). The HPLC system consisted of an X Terra ^TM^ column (Reversed C18, dimensions: 4.6 mm× 250 mm) containing 5 µm size adsorbent as stationary phase (Waters Corporation, Milford, Massachusetts, USA), L-7110 pump and L-7420 UV detector. The column was maintained at room temperature (25.0 ± 2.0 °C). Methanol 80% (v/v) was utilized as a mobile phase, flowing with a rate of 1.2 mL/min, and effluents were detected at 258 nm. TOL was eluted at 6 min under these conditions. A calibration curve was obtained in the concentration range of (2-14 µg/mL). The assay procedures were validated for linearity, accuracy and precision.

### In vitro antifungal activity

#### Fungal strain and inoculum preparation

A standard strain of Aspergillus niger (ATCC32656) was used in this study. The strain was cultivated on plates of Sabouraud Dextrose Agar (SDA) (Oxoid, Hampshire, UK) and incubated at 28 °C ± 2 for 48–96 h. The germinating spores were harvested in sterile normal saline solution, and the inoculum size was adjusted to 10^5^–10^6^ CFU/mL according to a predetermined correlation between optical density and the viable colony count.

#### Tested samples

Three treatments were tested for their in vitro antifungal activity; (i) the optimal TOL-PSRs formula (treatment A), (ii) TOL suspension containing 1 mg/mL (treatment B) and (iii) placebo solution (TOL-free optimal PSR formula).

#### Determination of the minimum inhibitory concentration (MIC) using microbroth dilution technique

MIC was determined according to the method described by Alastruey-Izquierdo et al. (Alastruey-Izquierdo et al., 2015), in accordance with the Clinical and Laboratory Standards Institute guidelines (Humphries et al., 2018). Two-fold serial dilutions of each of treatment A and B were prepared in double strength Sabouraud Dextrose broth (SDB) (500-0.24 µg/mL), then 10 μl of the spore suspension (inoculum size of 10^5^–10^6^ CFU/mL) were added to each well. Negative control (double strength SDB only) and positive control (double strength SDB and two fold serial dilution of placebo solution with 10 µL of the inoculum) were included. The microplates were incubated at 28 °C ± 2 for 48 h, and then the MIC was determined as the lowest concentration having no observable fungal growth. The test was performed in triplicates.

##### Determination of the minimum fungicidal concentration (MFC)

MFC for treatment A and treatment B was determined using broth microdilution method in accordance with the Clinical and Laboratory Standards Institute guidelines (Humphries et al., 2018). Briefly, after 24 h incubation of the 96-well plates containing the 2-fold serially diluted treatment A or B in double strength SDB together with the 10 μl spore suspension at 28 °C ± 2, 10 µL of the mixture of fungi and the two fold serially diluted treatment till the concentration of its MIC was spotted on SDA plates. The plates were incubated at 28 °C ± 2 for 48 h and the fungal count was determined and expressed as CFU/mL. The MFC was determined as the lowest concentration of treatment showing no fungal growth. The test was performed in triplicates.

### In vivo studies

#### Animals

The protocols for animal study were approved by the Research Ethics Committee at Faculty of Pharmacy, Cairo University, Egypt (PI 2982). A total of twelve male albino rabbits weighing 2 − 3 kg were included. Each rabbit was housed individually under appropriate storage conditions of temperature (25 °C ± 2), humidity and light/dark cycling. They were fed with standard dry food and tap water ad libitum. The animals were examined using a slit lamp to exclude any rabbit that has signs of ocular inflammation or disease.

#### Draize test

This test was performed to assess the irritancy of the tested formulations depending on a scoring system (Eldeeb et al., 2019). Three male albino New Zealand rabbits were used in this study. An aliquot of 100 µL of the optimal PSR formulation was dropped in the conjunctival sac of the right eye and saline was added into the left eye to be kept as control. The right eye was examined visually at intervals of 1, 2, 5, 8 and 24 h after installation for any irritation and scored according to Draize test (ElMeshad & Mohsen, 2016; Eldeeb et al., 2019). The scoring system ranged from 0 (no irritation) to +3 (highest irritation and redness).

#### In vivo histopathological study

Three male albino New Zealand rabbits were assigned in this test to evaluate the safety of the optimal TOL-PSRs formulation. One drop of TOL-PSRs was installed into the right eye while normal saline was dropped into the collateral eye as a control. The installation was repeated every hour for a period of 6 h (Yousry et al., 2020). The rabbits were then euthanized after anesthesia and their eyeballs were separated, washed with normal saline and fixed in 10% formaline in saline solution for 24 h. After that, the samples were washed using tap water then serial dilutions of alcohol (methyl, ethyl and absolute ethyl) were used for dehydration. Specimens were then cleared in xylene and embedded in paraffin at 56 degree in hot air oven for 24 h. Paraffin bees wax tissue blocks were prepared for sectioning at 4 microns thickness by a slidge microtome (Leica Microsystems SM2400, Cambridge, England). The obtained tissue sections were collected on glass slides, deparaffinized, stained by hematoxylin and eosin stain for routine examination using the light electric microscope (Bancroft et al., 1996).

#### Susceptibility test

Aspergillus niger (ATCC32656) was used as the test microorganism in the experiment. A parallel design of two groups each having three randomly chosen rabbits (total of six rabbits, *n* = 3 per group) was employed. Group I received the optimal TOL-PSR (treatment A) and group II received TOL suspension (treatment B). The experiment was performed as described before by Basha et al. (Basha et al., 2013), with slight modifications. Briefly, 50 µL (50 µg TOL) of each of treatment A or treatment B were installed in the lower conjunctival sac of the rabbit’s right eye using micropipette. In each rabbit, no treatment was applied in the left eye to serve as the control. At specific time intervals (2–24 h), two sterile filter paper disks (Whatman no. 5, 6 mm in diameter) were wetted by placing the disks under the eyelid of each eye for each rabbit. Then, the two disks of each eye were placed in a 1.5 mL Eppendorf tube containing 500 µL Sabouraud Dextrose Broth (SDB) inoculated with 10% v/v fungal spore suspension (10^5^–10^6^ CFU/mL). The other two disks were placed in a 1.5 mL Eppendorf tube containing 500 µL uninoculated SDB; to serve as a blank during the measurement of optical density. The tubes were then incubated at 28 °C ± 2 for 48 h under aerobic conditions. After incubation, 200 µL of each tube was transferred to a sterile 96-well plate and the optical density (OD_600nm_) was measured using an automated spectrophotometric plate reader (Biotek, Synergy 2, USA) at 600 nm. The obtained readings were expressed as average growth inhibition % (mean ± SD). The growth inhibition % was calculated using the following equation:

$$Growth\;inhibition\;\%=\frac{Control\;(left\;eye)\;OD\;600\;nm\;-Test\;(right\;eye)\;OD\;600\;nm}{Control\;(left\;eye)\;OD\;600\;nm}\times 100$$

## Results and discussion

### Statistical design analysis

Full factorial design is a valuable tool for evaluating the combined influence of different formulation variables on the characteristics of the prepared TOL PSRs using the least number of experimental runs. It allows for the investigation of all possible combinations of the levels of the selected variables in each complete trial or replication (Younes et al., 2018). A 3^1^.2^2^ full factorial design was used in this study and it was statistically analyzed through Design-Expert^®^ software. The selection of each factor levels was based on preliminary experiments and feasibility of preparing TOL PSRs at these values. The model selected was two factor interaction (2FI). The predicted R^2^ was calculated as measure of how good the model can predict a response value (Abdelbary et al., 2016). The adjusted and predicted R^2^ values are preferred to be close to each other to be in a reasonable agreement (Abd-Elsalam et al., 2018). Referring to the design analysis results (Table 3), it is worthy to note that the predicted R^2^ values are rationally matched with the adjusted R^2^ in all responses, which confirmed the adequate fitting of the selected model to the data. Furthermore, adequate precision measured the signal to noise ratio to ensure that the used model can be used to evaluate the design space (De Lima et al., 2011). A ratio greater than 4 is desirable and it was observed in all responses as shown in Table 3.

**Table 3.** Output data of the 3^1^.2^2^ factorial analysis of TOL-PSRs.

Responses	R^2^	Adjusted R^2^	Predicted R^2^	Adequate precision	Significant factors
EE%	0.9948	0.9914	0.9847	40.446	X_1_ (<0.0001), X_3_ (<0.0001)
PS (nm)	0.9567	0.9288	0.8726	18.869	X_3_ (<0.0001)
ZP (mV)	0.8274	0.7164	0.4928	9.298	X_3_ (<0.0001)

Abbreviations: EE%, entrapment efficiency percent; PS, particle size; ZP, zeta potential.

#### Effect of formulation variables on EE% of TOL-PSRs

**Figure 1.**
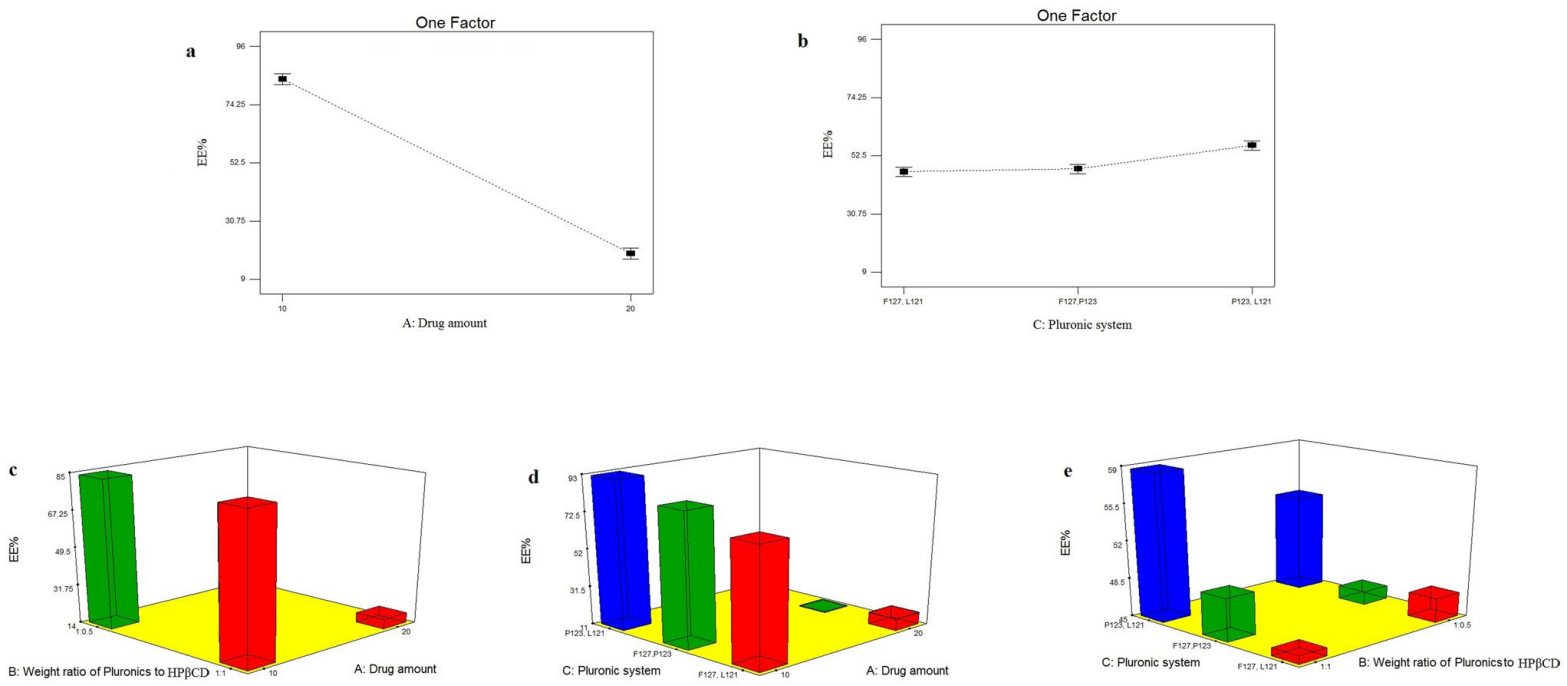
line plots of the significant effect of drug amount (X_1_)(a), Pluronic system (X_3_) (b), response 3-D plot for the combined effect of drug amount (X_1_) and weight ratio of Pluronics to HPβCD (X_2_) (c), drug amount (X_1_) and Pluronic system (X_3_) (d) and weight ratio of Pluronics to HPβCD (X_2_) and Pluronic system (X_3_)(e) on EE% of TOL PSRs.

The ability of PSRs to encapsulate significant amount of the drug is considered a key factor for optimum ocular delivery. EE% of the prepared TOL-PSRs ranged from 10.61 ± 0.86 (PSR 11) to 94.05 ± 1.91(PSR 3) (Table 2). The influence of drug amount (X_1_), weight ratio of Pluronics to HPβCD (X_2_) and Pluronic system (X_3_) on EE% of the prepared PSRs was graphically illustrated in Figure 1. EE% of the prepared PSRs was not significantly affected by varying weight ratio of Pluronics to HPβCD (X_2_) (*P* = 0.1559). On the other hand, ANOVA results showed that both drug amount (X_1_) and Pluronic system (X_3_) had significant effect on EE% (P<0.0001) (Table 3). Statistical analysis revealed that EE% had profoundly decreased by increasing drug amount. This could be related to the ability of low dose of TOL (10 mg) to saturate the inner core of PSRs and reaching the maximum loading efficiency as 94.05 ± 1.91 (PSR3) and 91.20 ± 0.71 (PSR6). By increasing drug amount, EE% decreased due to the rapid saturation of the inner core of the prepared PSRs which would lead to precipitation of the excess drug (Fares et al., 2018; Younes et al., 2018). It is obvious that PSRs can enhance EE% of poorly water soluble drugs to a certain extent after which increasing drug amount leads to decreased PSRs loading capability, drug precipitation and consequently low EE%. As for the effect of using different Pluronic systems on EE%, post hoc analysis showed that PSRs prepared using Pluronic system (P123 and L121) had the highest EE% compared to those prepared using the two other Pluronic systems (F127/P123 showed non significantly higher EE% compared to F127/L121 Pluronic system). This could be explained by that, the aqueous solubility of poorly water soluble drugs is inversely proportional with HLB values of the used Pluronic mixure (Salama & Shamma, 2015). Accordingly, the low ability of F127-containing PSRs to encapsulate TOL efficiently could be related to its higher hydrophilicity (HLB = 22) compared to P123 (HLB = 8) and L121 (HLB = 1). In another words, the abundance of hydrophobic Pluronics (P123/L121) provided a favorable medium for incorporation of poorly soluble drugs (Nour et al., 2016). The higher hydrophilicty of F127 could be also expressed by its larger number of hydrophilic PEO units (200) compared to low PEO number in both L121 (10) and P123 (40). These hydrophilic PEO units formed highly dense hydrophilic corona which considered as a steric shield which hinders the penetration of hydrophobic drugs like TOL into the core (Younes et al., 2018). Hence; TOL, being poorly water soluble drug, is expected to have higher affinity to P123/L121 mixtures compared to those containing highly hydrophilic F127. Furthermore, the formation of PSRs has been reported to occur preferably by inclusion of the hydrophobic PPO segments into CD cavities. Hence, P123/L121 Pluronic system is considered the most likely block to form complex with CD due to the higher number of PPO units compared to F127-containing Pluronic systems (Nogueiras-Nieto et al., 2009; Jansook et al., 2016). Furthermore, a slight interaction between drug amount (X_1_) and Pluronic system (X_3_) could be also observed from Figure 1D (*P* = 0.0006). At high level of X_1_ (20 mg) PSRs prepared using F127/P123 Pluronic system showed lower EE% compared to those prepared using F127/L121 Pluronic system. Hence, the effect of each factor on EE% was dependent on the other.

#### Effect of formulation variables on PS of TOL-PSRs

**Figure 2.**
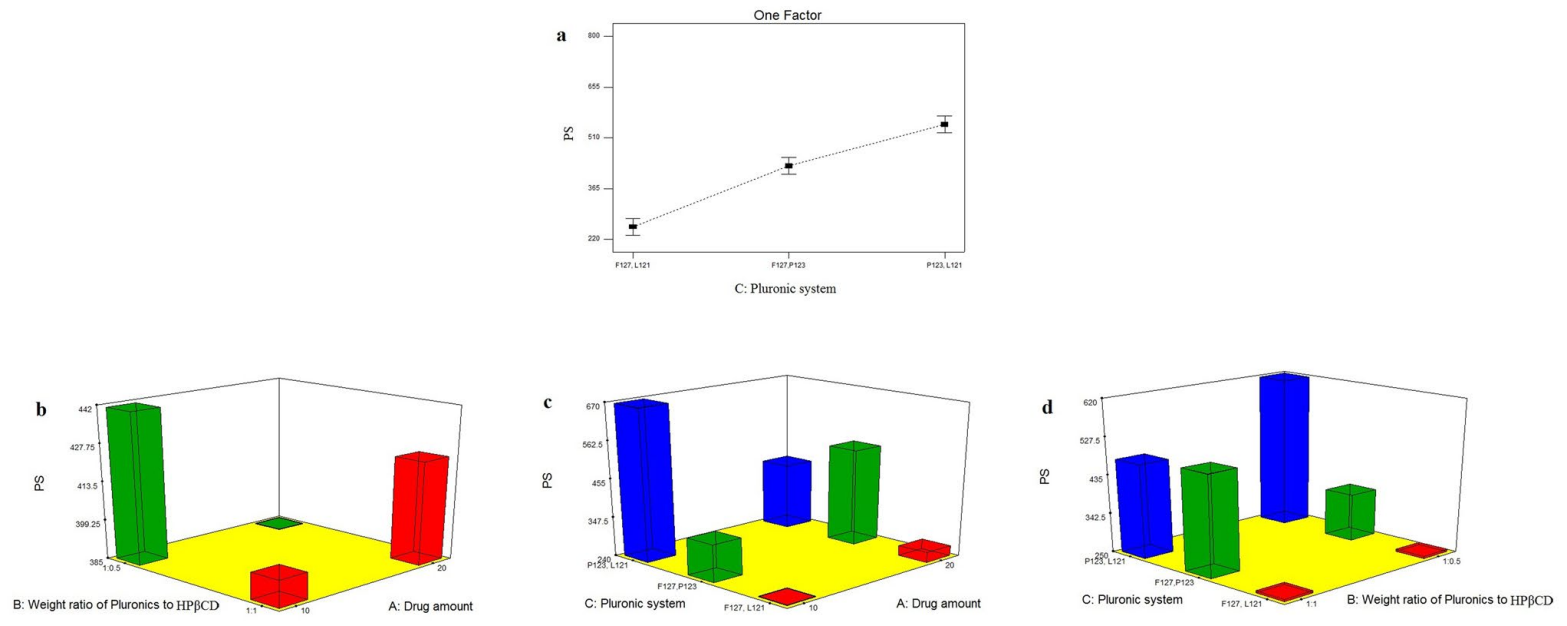
line plots of the significant effect of Pluronic system (X_3_) (a), response 3-D plot for the combined effect of drug amount (X_1_) and weight ratio of Pluronics to HPβCD (X_2_) (b), drug amount (X_1_) and Pluronic system (X_3_) (c) and weight ratio of Pluronics to HPβCD (X_2_) and Pluronic system (X_3_)(d) on PS of TOL PSRs.

PS values of all the prepared PSRs were in nano range (237.05 ± 12.80 to 773.65 ± 36.27 nm) as shown in Table 2. This small PS is a crucial to ensure adequate bioavailability, low irritation and good ocular tissue compatibility. The influence of drug amount (X_1_), weight ratio of Pluronics to HPβCD (X_2_) and Pluronic system (X_3_) on PS of the prepared PSRs is graphically illustrated in Figure 2. Based on the investigated design, only type of Pluronic system (X_3_) had significant effect on PS of the prepared PSRs (P<0.0001) (Table 3). Post hoc analysis showed that PS of TOL-PSRs came in the following order: F127/L121-based PSRs < F127/P123-based PSRs < P123/L121-based PSRs. This could be attributed to the significant higher amount of TOL entrapped within the hydrophobic core of P123/L121-PSRs which consequently resulted in PS enlargement (Younes et al., 2018). It is noticeable that F127/L121 based PSRs showed significantly smaller PS than those prepared using F127/P123. This could be attributed to the higher hydrophilicity of P123 (PEO = 40) compared to L121 (PEO = 10) which resulted in increasing water uptake with consequent increase in PS (Abdelbary & Aburahma, 2015). Furthermore, it has been reported that the addition of Pluronic F127, having long hydrophilic EO chains, to the more hydrophobic L121 resulted in the formation of small spherical mixed nanomicellar systems (Salama & Shamma, 2015). On the other hand, drug amount (X_1_) and weight ratio of Pluronics to HPβCD (X_2_) had no significant effect on PS of the prepared PSRs (*P* = 0.4614 and 0.8255, respectively). However, clear interaction had been observed between these two factors (*P* = 0.0340). At low drug amount (10 mg), PSRs prepared using 1:0.5 Pluronics to HPβCD showed significantly larger PS compared to those prepared using 1:1 weight ratio. On the other hand, at high drug amount (20 mg), PS significantly decreased by using 1:0.5 Pluronics to HPβCD. Furthermore, significant interaction had been also observed between X_1_ and X_3_ and between X_2_ and X_3_ (P<0.0001 and =0.0001, respectively). At high level of X_1_ and X_2_ (20 mg drug and Pluronics to HPβCD weight ratio of 1:1), F127/P123 Pluronic system showed larger PS than P123/L121. Moreover, PDI values of TOL-PSRs ranged from 0.32 ± 0.02 to 0.65 ± 0.10 as demonstrated in Table 2 which indicates that the prepared PSRs were polydisperse but within the acceptable range (0.3-0.7) (Danaei et al., 2018; Elsayed et al., 2020).

#### Effect of formulation variables on ZP of TOL-PSRs

**Figure 3.**
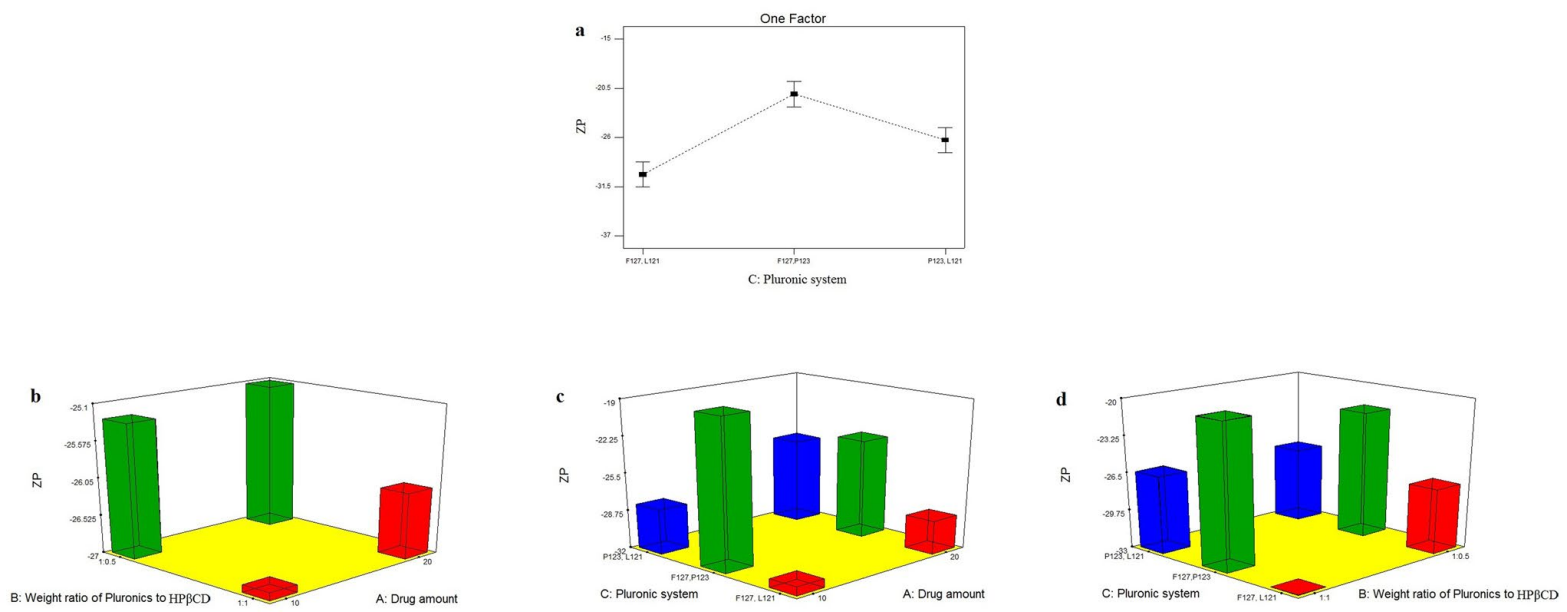
line plots of the significant effect of Pluronic system (X_3_) (a), response 3-D plot for the combined effect of drug amount (X_1_) and weight ratio of Pluronics to HPβCD (X_2_) (b), drug amount (X_1_) and Pluronic system (X_3_) (c) and weight ratio of Pluronics to HPβCD (X_2_) and Pluronic system (X_3_)(d) on ZP of TOL PSRs.

ZP is the measure of the net charge acquired by the surface of the dispersed PSRs and considered as an important indicator of the physical stability of nanosystems (Nour et al., 2016). The higher the electric charge adsorbed on nanoparticles surface, the stronger the repellant forces between particles which will prevent aggregation and give more stable dispersion (Honary & Zahir, 2013). As shown in Table 2, ZP of TOL-PSRs ranged from −18.80 ± 5.37 to −33.30 ± 4.53 mV. Since all formulations obtained negative ZP values, absolute values are used for discussion to prevent misperception. All TOL-PSRs formulae had negative charge due to the presence of ionizable thiocarbamate group in the structure of TOL which imparted a negative charge dominating over the neutral charge of the used non ionic Pluronics and HPβCD (Younes et al., 2018). The influence of drug amount (X_1_), weight ratio of Pluronics to HPβCD (X_2_) and Pluronic system (X_3_) on ZP of the prepared PSRs was graphically illustrated in Figure 3. Factorial analysis showed that only the type of the used Pluronic system (X_3_) significantly affected ZP of the prepared PSRs (P<0.0001) (Table 3). Post hoc analysis showed that F127/L121 based PSRs obtained the highest ZP values. ZP of nanoparticles is found to be size dependent. As the particle size decreases, a considerable increase in ZP is observed due to the increase in the particular surface area by decreasing PS which enhanced the screening efficiency of the charged solution around the small nanoparticle (Abbas et al., 2008). Hence, as previously mentioned under PS section, F127/L121 system formed significantly smaller PSRs compared to the other two systems which would increase the magnitude of the charge acquired by micellar surface. Oppositely, F127/P123 Pluronic system formed nanoparticles with the lowest ZP values. As known for Pluronics (tri block copolymer), the hydrophobic PPO segment anchors toward the hydrophobic surface while hydrophilic PEO chains extend in the aqueous phase and consequently cause outward shift of the slipping plane, where ZP is measured, to a point further away from the surface where the charge density is smaller than on the surface causing lower ZP values (Sis & Birinci, 2009; Abd-Elsalam et al., 2018). Hence, nonionic surfactant, based on their hydrophilicity (PEO content) could reside on the nano-dispersions surface leading to masking of their charge with resultant lower ZP values. Accordingly, F127 (PEO = 200)/L121 (PEO = 40) Pluronic system (being more hydrophilic compared to P123 (PEO = 40)/L121 (PEO = 10) one) could cause more shielding of the surface negative charge by moving the slipping plane to a point further out from the surface (where the charge density is smaller than on surface) resulting in significantly lower ZP values.

### Selection of the optimal TOL-PSRs formulation

Optimization of pharmaceutical formulation is generally aimed at tailoring the independent formulation variables for producing a robust high-quality product with optimal physico-chemical properties (Aziz et al., 2018). Hence, numerical and graphical analysis were applied using Design-Expert^®^ software and on the basis of desirability criterion to select the formula of choice from the prepared 12 formulations. The desirability constrains for the optimal TOL-PSRs formula (minimizing PS and maximizing EE% and ZP, as absolute value) were established in PSR1 with overall desirability 0.874. PSR1 was associated with the independent variables, namely, X_1_=10 mg, X_2_=1:1 and X_3_=F127/L121 and showed EE% of 71.55 + 2.90%, PS of 237.05 ± 12.80 nm and ZP of −32.65 ± 0.92 mV. To check the reasonableness of the optimization process, the observed and predicted responses of PSR1 were compared and the results are shown in Table 4. The observed values were in harmony with those predicted using Design-Expert^®^. Therefore, PSR1 can be considered as a promising novel nanoparticular formulation for ocular delivery of TOL and it was selected for further characterization.

**Table 4.** Predicted and observed values for the optimal PSRs (PSR1).

Response	Y_1_: EE%	Y_2_: PS (nm)	Y_3_: ZP (mV)	Desirability value
Observed values	71.55	237.05	−32.65	0.874
Predicted values	73.55	220.73	−34.21	
Percentage error	2.80	6.88	4.78	

Abbreviations: EE%, entrapment efficiency percent; PS, particle size; ZP, zeta potential.

### Transmission electron microscopy

**Figure 4.**
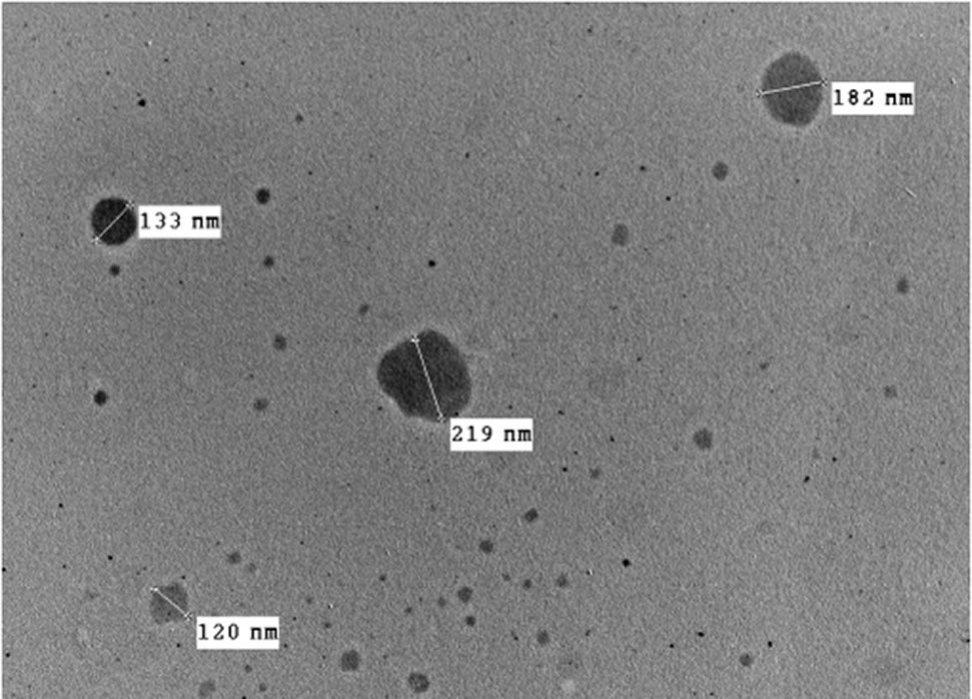
Transmission electron micrograph of PSR1.

TEM imaging is useful for assessing the results of Malvern particle size analyzer, as well as for describing the morphological features of the prepared system (Abd-Elsalam et al., 2018). Representative photomicrographs of PSR1 are illustrated in Figure 4. It is obvious that the developed pseudorotaxanes were spherical in shape with smooth surfaces and good dispersibility. Furthermore, the mean size of PSR1 was in a good harmony with the size previously obtained from Malvern particle size analyzer; in the range of 100-200 nm.

### Differential scanning calorimetry

**Figure 5.**
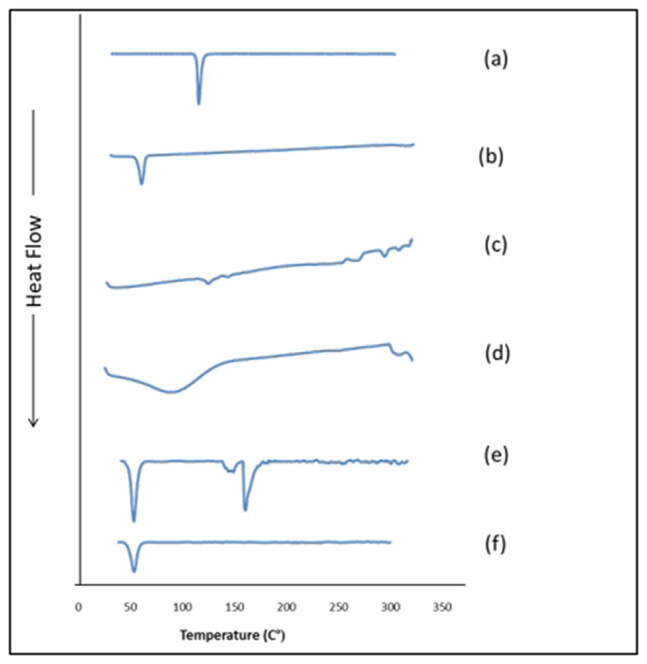
DSC thermograms of (a) TOL, (b) PF127, (c) PL121, (d) HPβCD, (e) physical mixture of TOL-PSRs and (f) PSR1.

DSC is a convenient tool for assessing the compatibility between the drug and the used excipients and for evaluating their physical nature (Emad Eldeeb et al., 2019). Figure 5 represents the thermograms of pure TOL, Pluronic F127, Pluronic L121, HPβCD, physical mixture of TOL with PSR1 components and the lyophilized PSR1. The DSC scan of pure TOL depicted a sharp single endothermic peak at 112.31 °C corresponding to its melting point (Kumari et al., 2017). This sharp peak also indicated the crystalline nature of TOL. The thermogram of Pluronic F127 showed sharp endothermic peak corresponding to its melting point at 56.60 °C (Younes et al., 2018). Concerning therrmogram of PL121, small endothermic peak was detected at 121.43 °C indicating its boiling point (Nour et al., 2016). HPβCD showed a very broad endothermic transition, which reached the maximum at 86.15 °C. This was attributed to the release of the bound water in its cavity (George & Vasudevan, 2012). The sharp endothermic peak of TOL was conserved in the thermogram of the physical mixture, but disappeared in that of the lyophilized optimal psudorotaxane formulation (PSR1) confirming the transformation of TOL from crystalline to amorphous form due to its complete dispersion in the prepared nano-micellar system (Sayed et al., 2018). The thermograms of the physical mixture and PSR1 showed an endothermic peak at 53.77 and 46.89, respectively representing the fused peaks of PF127 and P121 (Younes et al., 2018).

### Comparison of the measured responses of the optimal TOL-PSRs formulation and conventional PMMs

The measured responses (EE%, PS and ZP) of PSR1 (the optimal pseudorotaxanes) and the conventional PMMs are shown in Table 2. Comparing EE% of both nano-systems, EE% of PSR1 was significantly higher than the conventional PMMs (*P* = 0.013) due to the combined use of polaxmers and HPβCD which was clearly more effective in enhancing the drug aqueous solubility compared to the corresponding drug/polymer and drug/CD binary system. The addition of polaxmer and CDs resulted in decreasing drug crystallinity and synergistically enhancing the solubilizing action of both of them (Folch-Cano et al., 2014). These results came in agreement with the observation made by Hirlekar et al. who showed that the solubility of irbesartan was enhanced in the presence of βCD together with water soluble polymers (Hirlekar et al., 2009). It was also shown that the addition of polymers to drug/CD complex can aid in amorphization and PS reduction with further improvement in the dissolution rate of the complex (Hirlekar et al., 2009). PSR1 showed numerically but not significantly smaller PS compared to the conventional micellar formulation (*P* = 0.751). There was no significant difference in ZP of PSR1 when compared to conventional micellar formulation (*P* = 0.185).

### In vitro release

**Figure 6.**
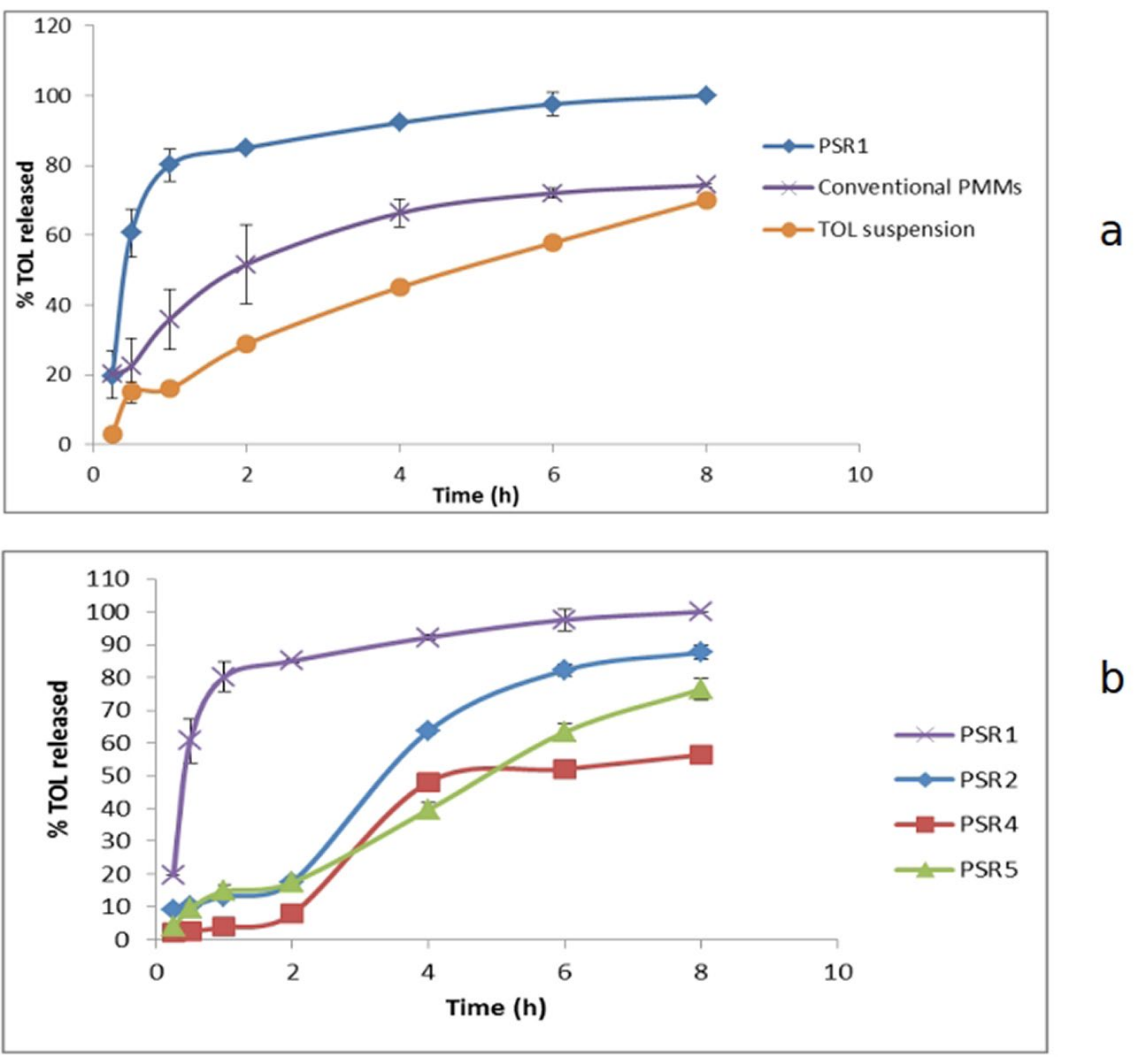
In vitro release profile of TOL from PSR1 and conventional polymeric mixed micelles (PMMs) (a) and the release profiles of TOL from the three selected formulae (PSR2, PSR4 and PSR5) and PSR1 (b).

The drug release from the PSR1 was studied and compared to the conventional PMMs and drug suspension in terms of release rate and extent as demonstrated in Figure 6a. It was obvious that complete release of the drug was achieved from PSR1 by the end of experiment (8 h). During the same time period, only 74.30% and 70.04% of the drug was released from conventional polymeric micelles and drug suspension, respectively. Furthermore, T_50%_ was shorter for PSR1 (0.5 h) when compared to TOL polymeric micelles (2 h) and drug suspension (5 h). This could be attributed to the enormous surface area of the formed nanoparticles and the incorporation of HPβCD which improve the apparent drug solubility and dissolution (Emad Eldeeb et al., 2019). The co-existence of HPβCD and poloxamer mixture in pseudorotaxane construct could enhance drug release rate and extent by acting as dissolution enhancers (Sayed et al., 2018). These results came in agreement with the observation reported by Zhang et al. who showed that complete release of theophylline could be achieved through the dissociation of theophylline/HPαCD/PEG polyrotaxane supramolecular structure (Zhang & Ma, 2013). Also Hirlekar et al. showed that 99.69% of irbesartan was released from drug/βCD/PVP K-90 ternary system compared to 70.63% from irbesaratn/βCD binary system and 21.86% from plain irbesartan which confirmed that the complexation and solubilizing efficiency of CD increased in the presence of PVP K-90 (Hirlekar et al., 2009). Moreover, the release profiles of PSR1, conventional micellar formulation and drug suspension were best fitted to Higuchi-diffusion model (highest R^2^, Table 5) which confirmed that drug transport out of nanomicelles was mainly driven by diffusion-controlled mechanism. Regarding the release profiles of TOL from the three selected formulae (PSR2, PSR4 and PSR5) and PSR1(Figure 6b), ANOVA results revealed that changing the used Pluronic system and Pluronic system to HPβCD weight ratio significantly affected the rate (T_50%_) and extent (Q8h) of TOL release. It was shown that using Pluronic system to HPβCD weight ratio 1:1 significantly increased the rate and extent of TOL release compared to 1:0.5. The formation of PSR usually needs a great number of CD molecules per Pluronic copolymer chain (Nogueiras-Nieto et al., 2009). Hence, using 1:1 weight ratio of Pluronics to HPβCD is the optimum ratio for complete coverage of the hydrophobic PPO segments and formation of larger number of PSRs supramolecular structure and consequently caused significantly higher rate and extent of drug release compared to using 1:0.5 weight ratio. Considering the used Pluronic system, formulae prepared using Pluronic system F127/L121 showed significantly higher release rate and extent compared to those prepared using Pluronic system F127/P123. This could be related to that P123 has a higher molecular weight compared to L121 with consequent more abundance of O and OH points which enhance the attachment of the drug molecules through hydrogen bonds resulting in slower release rate (Nour et al., 2016).

**Table 5.** Fitting PSR1, conventional PMMs and drug suspension release data to zero, first, and Higuchi diffusion models.

Formula code	Zero order	First order	Higuchi diffusion	Best fitting model
PSR 1	0.5488	0.3874	0.6901	Higuchi diffusion
Conventional PMMs	0.8537	0.7576	0.9518	Higuchi diffusion
Drug suspension	0.9708	0.6934	0.9908	Higuchi diffusion

Abbreviations; PSR1, the optimal polymeric pseudorotaxans; PMMs; polymeric mixed micelles.

### Ex vivo corneal permeation

**Figure 7.**
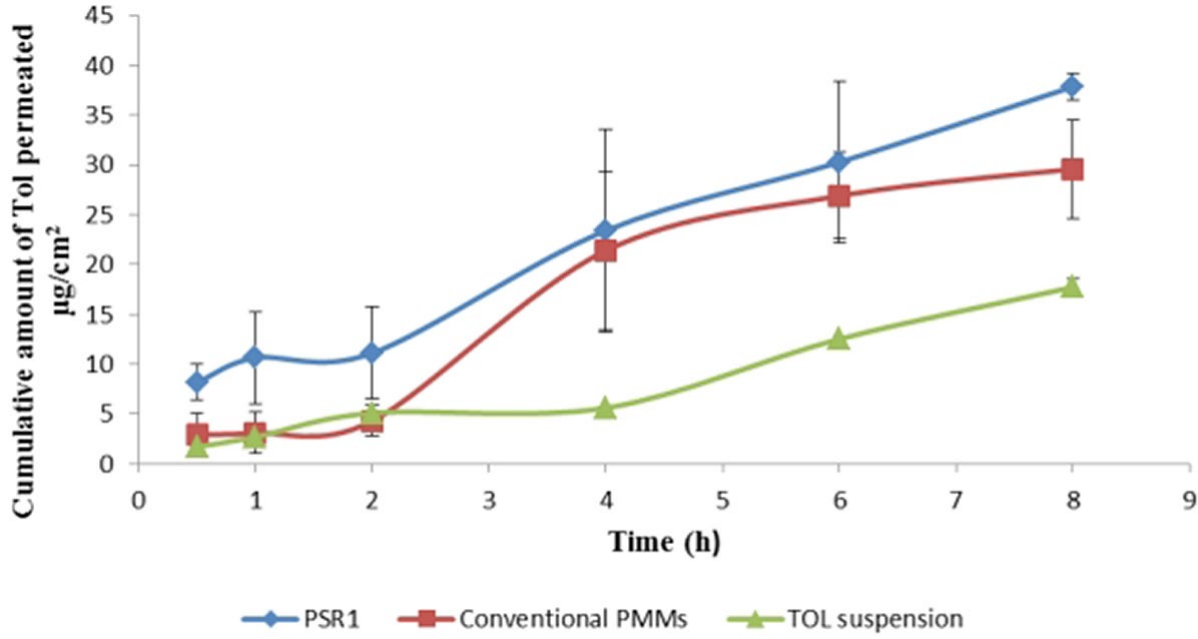
Cumulative amount of TOL permeated per unit area across excised corneas via PSR1 compared to conventional PMMs formulation and drug suspension.

The permeability of TOL from PSR1 was studied using excised rabbitsʼ corneas and its permeation profile was compared to that obtained from TOL conventional PMMs and drug suspension as shown in Figure 7. It is obvious that TOL permeation increased in the order of PSR1> conventional polymeric micelles > drug suspension. Comparing the corneal permeability parameters, both PSR1 and TOL-PMMs significantly increased the drug flux and resulted in higher amount permeated per unit area in 8 h compared to drug suspension (P<0.05) (Table 6). The ER was more than 2 for PSR1 and more than 1.5 for TOL PMMs compared to drug suspension. This significant enhanced TOL ocular delivery through PSRs and conventional nano-micelles could be related to the penetration enhancing capability of the Pluronics included in their constructs which enhanced the ocular mucosal membrane permeability through loosening the tight junction in the corneal epithelial barriers, facilitating TOL penetration through paracellular route. Furthermore; using these polymeric vehicles could impart a slight lowering in ocular surface tension and increased tear film viscosity which facilitate drug mixing with tear film and consequently its penetration (Malhotra & Majumdar, 2001). Besides, the nanometric size of PSRs and mixed micellar formulation facilitated their passage as a fine dispersion through the hydrated narrow corneal stromal network compared to coarse PS of drug suspension (Zhou et al., 2017). Furthermore, the results also showed the superiority of PSR1 over conventional TOL polymeric micelles. This could be attributed to the presence of HPβCD in PSRs constructs which acts mainly as solubilization enhancer and consequently caused significant higher EE% compared to conventional polymeric micelles which resulted in increased TOL concentration at the precorneal tear film with resultant increase in its absorption rate (Malhotra & Majumdar, 2001). Additionally, CDs, being large hydrophilic molecules, can enhance the permeation of lipophilic drugs like TOL through ocular surface by effectively shrinking and decreasing the effective thickness of the unstirred water layer (tear fluid layers) which covers the eye surface and it is considered as the main barrier against ocular delivery of lipophilic drugs (Muankaew & Loftsson, 2018).

**Table 6.** Corneal permeability parameters of PSR1 compared to conventional PMs and drug suspension.

Formulation	**Total amount of drug permeated per unit area in 8 h(µg/cm^2^)** ^a^	**J_max_(µg/cm^2^/h)** ^a^	**ER**
PSR1	37.87 ± 1.35	7.40 ± 0.26	2.13
Conventional PMMs	29.60 ± 4.94	5.87 ± 0.96	1.67
Drug suspension	17.74 ± 0.89	3.46 ± 0.17	1

^a^ Data presented as mean ± SD (*n* = 3).

Abbreviations: J_max_, flux; ER, enhancement ratio; TOL-PSRs, tolnaftate pseudorotaxans; PMMs, polymeric mixed micelles.

### In vitro antifungal activity

#### Determination of the minimum inhibitory concentration

Antifungal assays were performed using microbroth dilution technique to determine whether the optimal PSR influences TOL antifungal activity. The MIC of PSR1 (treatment A) was 0.49 µg/mL, while TOL suspension (treatment B) showed a higher MIC (1.95 µg/mL) and that for the placebo solution was > 500 µg/mL. Hence, the antifungal activity of PSR1 was significantly enhanced compared to those of TOL suspension. This could be attributed to the enhanced drug solubility in PSR1 which aided in its fungal cell wall penetration and consequently inhibition of ergosterol biosynthesis which is responsible for its antifungal activity (Li et al., 2018; Sayed et al., 2018).

##### Determination of the minimum fungicidal concentration (MFC)

MFC was determined using broth microdilution for both treatment A and B, where both PSR1 and TOL suspension showed fungicidal effect after incubation at 28 °C ± 2 for 48 h. PSR1 (treatment A) showed more pronounced fungicidal effect at 3.9 µg/mL (8x MIC), while MFC of TOL suspension (treatment B) was higher with a value of 125 µg/mL (64x MIC). Hence, the fungicidal activity of TOL suspension was inferior to that of PSR1.

### In vivo studies

#### Draize test

Draize test is the most reliable test to assess the safety of the ocular formulations (Eldeeb et al., 2019). The obtained results showed that PSR1 didn’t cause irritation on Draize scale of 0 to + 3 after 24 h when compared to control eye.

#### In vivo histopathological studies

**Figure 8.**
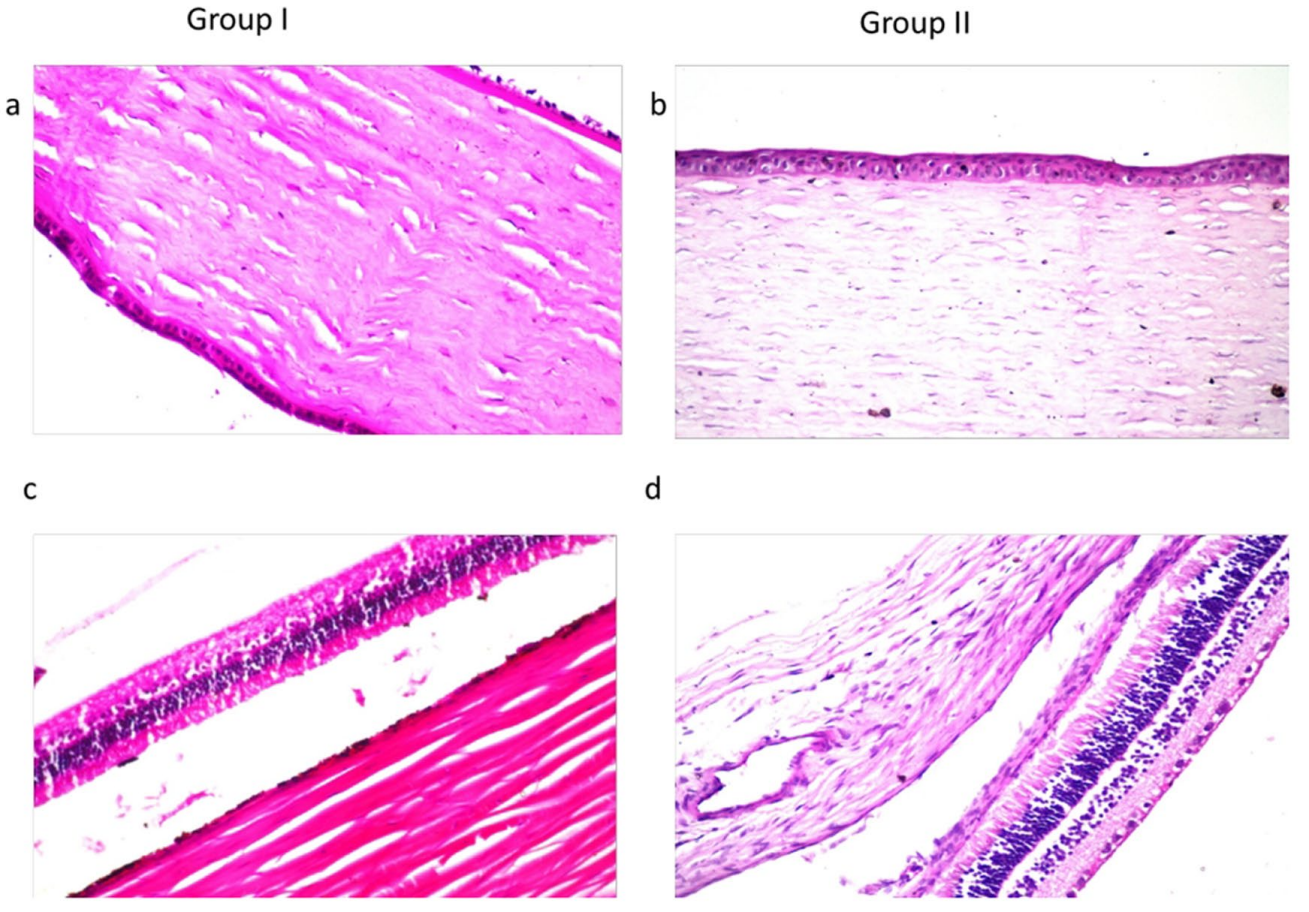
Photomicrographs showing histopathological sections (hematoxylin and eosin stained) of normal untreated rabbit eye (groupI) and rabbit eye treated with PSR1 (group II); where a and b represent the cornea showing normal histopathological structure of corneal epithelium, stroma and endothelium at magnification power of x40, while c and d represent the retina choroid and sclera with normal histopathological structure at the same magnification power.

The examination of photomicrographs showed no histopathological alteration in the cornea (including the lining corneal epithelium, the underlying stroma and endothelium) (Figure 8b) as well as the retina, choroid and sclera (Figure 8d) when compared to the control group (Figure 8a and c). Therefore, PSR1 is well-tolerated system and can be safely applied to the eye.

#### Susceptibility test

**Figure 9.**
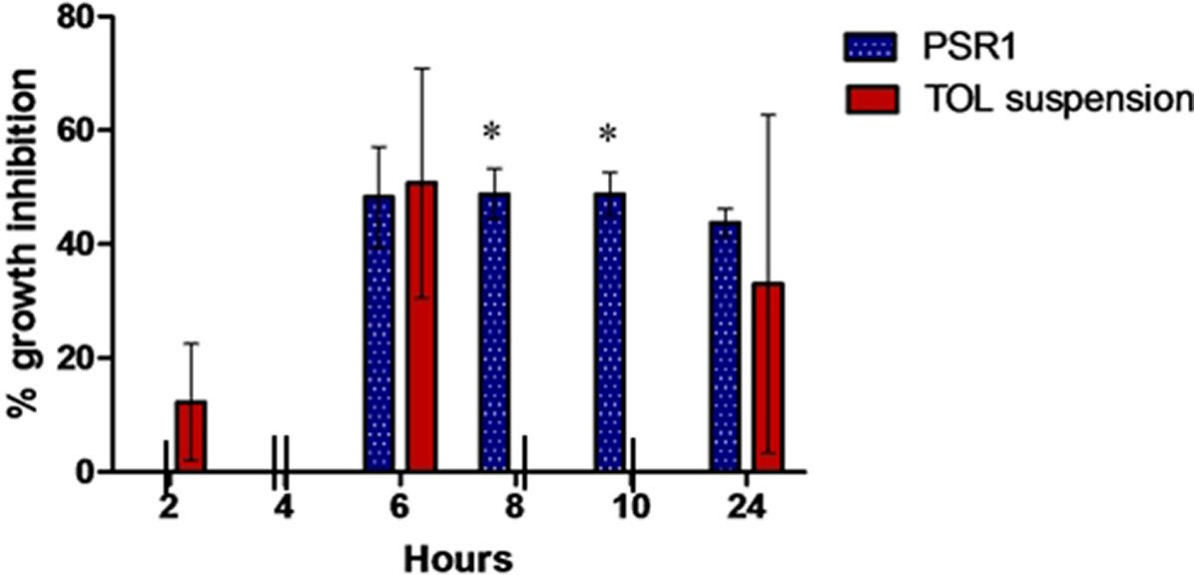
Percentage of growth inhibition of *Aspergillus niger* (ATCC32656) by PSR1 (treatment A) compared to TOL suspension (treatment B) in rabbit external ocular tissue. Data represent the means of % growth inhibition after time interval (2 – 24 h) of administration ± SD, *n* = 3 (one-way ANOVA, *p*-value < 0.05). (*) Means statistically significant difference exists between columns. ( ) indicates no bar (0 % growth inhibition). The chart was generated using GraphPad Prism (v5).

The percentage growth inhibition of Aspergillus niger was related to the drug’s retention time on the eye surface following topical administration (Figure 9). The percentage inhibition of PSR1 formula reached its maximum level (48.3 ± 8.76%) 6 h after administration till 24 h. TOL suspension reached its maximum inhibition (50.75 ± 20.15%) after 6 h of administration as well but it decreased remarkably to reach no inhibition after 8 h of administration till 24 h which reached 27 ± 38.18%. The percentage growth inhibition of PSR1 was significantly higher than that of TOL suspension after 8 h (*P* = 0.004) and 10 h (*P* = 0.002) of administration (one way ANOVA, *P* < 0.05).

PSR1 sustained the antifungal activity of TOL on the ocular surface for a relatively longer time compared to TOL suspension with an area under the curve 2.97 folds higher than that of the suspension (AUC_2h-24h_ = 890.6 and 300.3, respectively). This could be attributed to the smaller PS of PSR1 compared to drug suspension, resulting in longer residence time and better corneal contact of TOL (Basha et al., 2013; Fahmy et al., 2021). Therefore, PSR1 could be a promising alternative to the conventional eye drops due to its sustained effect.

## Conclusion

In this study, polymeric pseudorotaxanes (PSRs) were fabricated using film hydration technique for ocular delivery of tolnaftate (TOL). A 3^1^.2^2^ factorial design was employed to study the effect of different formulation variables on PSRs characteristics and to select the optimal PSRs (PSR1). PSR1 showed spherical morphology using TEM. Moreover, it was subjected to DSC studies which confirmed the internalization of TOL within PSR structure. In addition, a comparative study was conducted between PSR1 and conventional polymeric mixed micelles (PMMs) which confirmed the superiority of PSR1 in release rate and extent compared to conventional PMMs and drug suspension. Also PSR1 significantly increased the ex vivo corneal flux compared to drug suspension In vitro antifungal studies were performed and confirmed that TOL has strong fungicidal activity compared to drug suspension. Furthermore, the in vivo Draize test and histopathological studies confirmed the non-irritancy of PSR1. The fungal susceptibility of TOL using Aspergillus niger (ATCC32656) showed that PSR1 has superior residence on the eye surface compared to drug suspension. Therefore, PSRs could be considered as a novel ocular drug delivery system for delivering wide range of practically insoluble drug with high safety and efficiency.
